# Biomass-derived Carbon Quantum Dots for Visible-Light-Induced Photocatalysis and Label-Free Detection of Fe(III) and Ascorbic acid

**DOI:** 10.1038/s41598-019-49266-y

**Published:** 2019-10-21

**Authors:** Gouri Sankar Das, Jong Pil Shim, Amit Bhatnagar, Kumud Malika Tripathi, TaeYoung Kim

**Affiliations:** 1 Department of Bionanotechnology, Gachon University, 1342 Seongnam-daero, Sujeong-gu Seongnam-si, Gyeonggi-do, 13120 South Korea; 2 Department of Materials Science and Engineering, Korea University, 145 Anam-ro, Seongbuk-gu, Seoul 02841 Korea; 3 Department of Environmental and Biological Sciences, University of Eastern Finland, FI-70211 Kuopio, Finland; 4 Department of Materials Science and Engineering, Gachon University, 1342 Seongnam-daero, Sujeong-gu Seongnam-si, Gyeonggi-do, 13120 South Korea

**Keywords:** Nanoscale materials, Materials science

## Abstract

Visible-light-driven photocatalysts prepared using renewable resources are crucial but challenging to develop for the efficient degradation of organic pollutants, which is required to solve ever-increasing water deterioration issues. In this study, we report a visible-light-responsive photocatalyst for the efficient degradation of methylene blue (MB) as a model pollutant dye. Green-emissive carbon quantum dots (CQDs) were synthesized from pear juice via a facile, scalable, one-pot solvothermal process. The as-synthesized CQDs exhibit superior photocatalytic activity under visible-light irradiation owing to their efficient light absorption, electron transfer, and separation of photogenerated charge carriers, facilitating ~99.5% degradation of MB within 130 min. A possible mechanism for the photocatalysis is proposed on the basis of comprehensive active species trapping experiments. Furthermore, the CQDs were used in a specific sensitive assay for Fe(III) and ascorbic acid (AA), even with interference from other metal ions. The fluorescence emission of CQDs was “turned off” specifically upon binding of Fe(III) and “turned on” with AA. The prepared CQDs represent efficient photocatalysts and fluorescent probes that are not restricted by toxicity, cost, or lack of scalability.

## Introduction

There is an urgent demand for the wastewater remediation because of the severe water scarcity arising with ever-increasing populations. With the development of textile industries and other environmentally unfriendly human activities, contamination of water with toxic organic dyes is a common cause of environmental damage^1^. Discharge of chemically stable and non-biodegradable toxic organic dyes into aquatic environments is a growing problem because of their highly toxic nature^1,2^. Organic dyes can affect the oxygen dissolution, sunlight penetration, and transparency of water, and can also reduce photosynthesis in water bodies, even when present at very low concentrations (~10 ppm)^3,4^. In addition, some dyes are highly mutagenic and carcinogenic to living organisms, including humans^4^. Efficient and low-cost techniques therefore need to be designed and developed for the removal of toxic dyes from industrial wastewater.

Among diverse physical, chemical, and biological techniques, visible-light photocatalysis has become prominent in water remediation in the past few years because it is environmentally benign and mild in nature, generates negligible by-products, and has high functional-group compatibility^5–8^; these benefits are particularly strong for photocatalytic degradation of soluble dyes, making it the most advanced method^6,7,9^. Since the first photocatalytic use of TiO_2_^10^, semiconductor photocatalysis has attracted considerable research attention; however, the typically employed hybrid photocatalysts are toxic, less active in the visible region, expensive, or require time-consuming and multi-step synthesis procedures, limiting their practical applications^11–13^. Much effort has been dedicated to developing novel photocatalyst systems to maximize the utilization of visible light^14^. Photocatalytic materials with high photocatalytic performance in the visible region are therefore highly desirable.

Nanocarbons and their hybrid materials have attracted extensive attention in their own right^15^. Among the various nanocarbons, carbon quantum dots (CQDs) are particularly attractive because of their visible-light absorption and fascinating physicochemical properties^16–18^. CQDs, an emerging class of zero-dimensional carbonaceous nanomaterials less than 10 nm in size, are attractive alternatives to semiconductor photocatalysts as they offer strong photoresponsiveness, redox properties, stability, nontoxic characteristics, and facile tunability^16^. CQDs typically consist of a heterogeneous distribution of sp^2^ and sp^3^ carbon domains with heteroatomic functional groups, which create discrete energy levels within a CQD leading to unique photophysical, chemical, and electrical properties^19^. Notably, CQDs possess electron-accepting, electron-transfer, and electron-transport properties, which make them promising agents for light harvesting^20^. Their ease of synthesis and modification, high aqueous solubility, and edge effects have led to them being widely used in photomediated applications and sensing technologies ranging from biomarker sensing^21^ and detection of heavy metal ions^22^, DNA^23^, and biomolecules^24^, to hydrogen generation^11,25^, CO_2_ reduction^26^, and dye degradation^16,27^. Because of their electron-transfer and light-harvesting properties, CQDs have mostly been used as additives for photocatalysts^28–30^, while they have not been much explored for visible-light-induced photocatalysis due to their limited photocatalytic activity under visible light.

In this work, the potential of CQDs to be used as photoluminescent (PL) probes is explored based on their “on-off” emissions for the efficient and selective detection of Fe(III) and ascorbic acid (AA). The fabrication of these PL probes has potential significance owing to their simple, real-time, and economic strategy with high accuracy^31,32^. Although metal ions are crucial for life and the environment, they are toxic to living organisms, including humans, at elevated concentrations^31–33^, so the detection of metal ions is important. The selective detection of Fe(III) is especially vital as it is one of the most essential elements for biological processes and living organisms^34^. For example, the deficiency and surplus of Fe(III) can induce various disorders including hemochromatosis, diabetes, liver damage, heart failure, anemia, and Parkinson’s disease^34,35^. Fe(III) is also a common water pollutant^36^. However, practical detection of Fe(III) with PL probes is limited due to cross-sensitivity with common co-existing Al(III), Cu(II), and Cr(III) ions^37^. Therefore, developing methods to selectively monitor Fe(III) in an accurate and facile manner is important. Similarly, AA, an important micronutrient, plays a range of roles in biological systems, including as an antioxidant, a cofactor in essential enzymatic reactions, and a free-radical scavenger, and it is also involved in gene expression and cell division^38–40^. AA is known to be a medication for drug poisoning, cardiovascular disease, scurvy, liver disease, gout, allergic reactions, diabetes, and atherosclerosis^38,39,41^. The detection of AA in medical assays and diagnosis is therefore quite significant for human health but also challenging due to cross-sensitivity with dopamine and uric acid^40^. To the best of our knowledge, the direct use of CQDs as a PL probe for selective Fe(III) sensing and subsequent AA detection has not been previously reported.

Herein, green fluorescent CQDs were synthesized through hydrothermal treatment of pear juice in an one-step process. The CQDs exhibited excellent photostability even in the presence of various interfering ions at high concentrations. The as-synthesized CQDs possess excellent visible-light-induced photocatalytic activity towards dye degradation, achieving 99.5% degradation of MB within 130 min. More importantly, we have found that the CQDs can be used as fluorescent “nanoprobes” for the selective detection of Fe(III) and AA based on PL “turn-off” and “turn-on” mechanisms. The PL emission of the CQDs can be strongly suppressed by Fe(III) and selectively restored by AA with a detection limit of 1.27 µM.

## Experimental Section

### Materials

Laboratory-grade reagents were acquired from commercial suppliers and used without any further processing or purification. Except for K_2_Cr_2_O_7_ and Al(NO_3_)_3_·9H_2_O, all metal ions used in this study were chloride salts and procured from Alfa Aesar (South Korea). MB, p-BZQ, t-BA, and Na_2_EDTA used in the photocatalytic experiments were acquired from Sigma Aldrich. Ascorbic acid, citric acid, dopamine, uric acid, glucose, cytosine, thymine, guanine, and adenine were purchased from Sigma Aldrich, Korea. Millipore water (18.2 MΩ cm at 25 °C) was used for the preparation of all aqueous solutions.

### Instrumentation

The size and morphology of the as-synthesized CQDs were analyzed using transmission electron microscopy (TEM) and high-resolution TEM (FEI Tecnai F30, operated at 300 kV). A Bruker Vector 22 spectrometer was used to record the Fourier transform infrared (FTIR) spectra of CQDs in the range of 400–4000 cm^−1^. A ULVAC-PHI X-tool X-ray photoelectron spectrometer was used to examine the surface properties of the CQDs using an Al K_α_ X-ray source. The photoluminescence (PL) emission measurements were acquired at room temperature with a Varian fluorescence spectrometer. PL mapping images of the CQDs were acquired with a Leica inverted microscope (Leica DM 2500, Leica Microsystems Ltd.) Fluorescence decay spectrum was recorded with a EasyLife II fluorometer system. UV-vis absorption measurements were carried out with a Varian 50 Bio UV-vis spectrophotometer.

### Synthesis of CQDs

CQDs were synthesized by a facile hydrothermal treatment of pear juice. Filtered fresh juice was poured into a 100 mL stainless-steel autoclave with a Teflon lining and thermolyzed for 36 h at 180 °C. The oven was cooled to room temperature. The CQDs were collected by filtration with a 0.22 µm membrane filter as an orange-brown highly viscous liquid.

### Photocatalytic activity measurements

The photocatalytic activity of the CQDs was evaluated by monitoring the degradation of MB as a model organic pollutant. In a typical photodegradation experiment, 200 µL of CQDs were added into 50 mL of MB solution (45 µM) and stirred for 30 min in the dark to achieve adsorption equilibrium. The container was then sealed and exposed to visible light from a 60 W tungsten lamp with a distance between the light source and the samples of 10 cm. At the 10 min of intervals, aliquots were taken out and placed in quartz cells for UV-vis absorbance measurements to determine the remaining concentration of MB using the Langmuir-Hinshelwood equation, log(C_o_/C) = kt. C_o_ is the initial concentration of MB prior to light illumination, C is concentration of MB at time t, and k is the rate constant. Experiments were carried out in triplicate in an air-conditioned room to prevent heat effects.

### Fluorescence “turn-off-on” assay for sensing Fe(III) and AA

Detection of Fe(III) and AA were performed at room temperature in aqueous solution. The control sample of CQDs was prepared by adding 100 µL of CQDs to 10 mL of water. In a typical assay, 10 µL of an aqueous solution of Fe(III) (1 × 10^−3^ M) was mixed with 2 mL of the CQD solution and the PL intensity was recorded after 1 min at an excitation wavelength of 420 nm and a constant excitation/emission slit width of 10 nm. The selectivity to Fe(III) was investigated in triplicate with different metal ions (Ag(I), Al(III), Ba(II), Ca(II), Cd(II), Co(II), Cu(I), Cu(II), Cr(II), Cr(III), Cr(VI), Fe(II), Hg(I), K(I), Mn(II), Na(I), Ni(II), Pb(II), and Zn(II)) in a similar way.

The detection of AA was performed by the addition of an aqueous solution of AA (1 mM) to the quenched CQDs/Fe(III) system with the PL intensity measured after 2 min. The interference of different biomolecules towards AA was investigated in the presence of CA, DA, UA, glucose, Cy, Th, Gu, and Ad under similar experimental conditions.

## Results and Discussion

### Synthesis and characterization of CQDs

**Figure 1:**
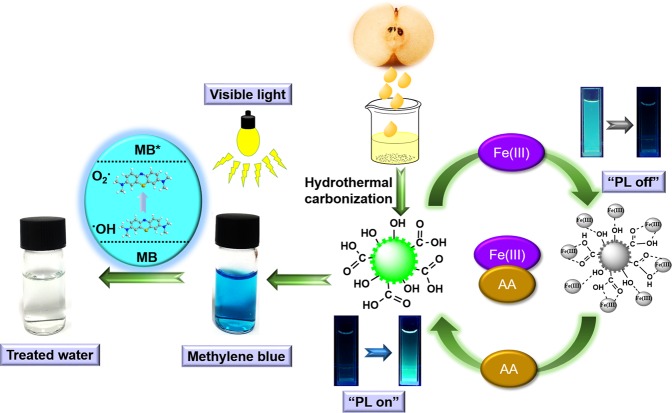
Schematic of the one-step synthesis of carbon quantum dots (CQDs) from pear juice and their visible-light-induced dye degradation and sensing applications.

CQDs were synthesized by direct hydrothermal treatment of pear juice at 180 °C for 36 h and then collected by filtration with a 0.22 µm membrane. The use of pear juice as a natural precursor and low synthesis temperature allowed for the highly scalable and sustainable synthesis of CQDs. The as-synthesized CQDs were utilized for the aqueous-phase photocatalytic degradation of MB and selective sensing of Fe(III) and AA as shown in Fig. 1.

**Figure 2:**
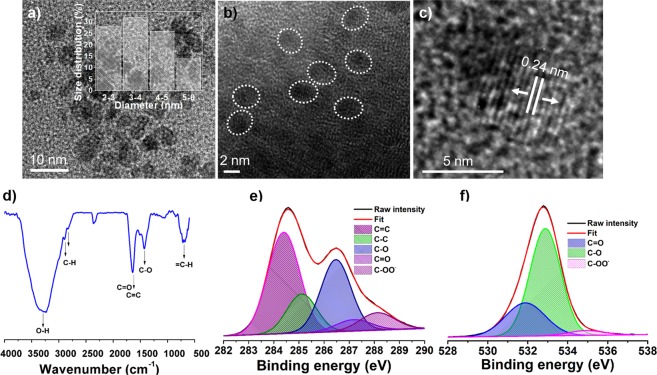
Morphological and microstructural analysis of CQDs. (**a**) TEM image of CQDs at low magnification, inset: size-distribution histogram; (**b**) HR-TEM image of CQDs showing their nearly spherical morphology; (**c**) HR-TEM image of a CQD showing graphitic spacing; (**d**) FTIR spectrum. High-resolution XPS spectra of the deconvoluted (**e**) C 1 s peak and (**f**) O 1 s peak.

The morphology and microstructure of the CQDs were investigated using transmission electron microscopy (TEM) and high-resolution TEM (HR-TEM) analysis. Typical TEM image in Fig. 2(a) and HR-TEM image in Fig. 2(b) show that the CQDs were nearly monodisperse and almost spherical. The CQDs exhibited a narrow size distribution in the range of 3–6 nm as calculated from a statistical analysis of 100 CQDs (Inset of Fig. 2(a)). The HR-TEM image in Fig. 2(c) shows that the CQDs were highly crystalline with a d-spacing of 0.24 nm^42^.

The Fourier transform infrared (FTIR) spectrum in Fig. 2(d) shows the presence of significant surface functional groups. The merged peaks at ~1635 cm^−1^ were assigned to C=O and C=C stretching in the conjugated structure. The C=O and C-O stretching vibrations at ~1635 cm^−1^ and ~1424 cm^−1^, respectively, indicate the presence of oxygenated carboxyl and hydroxyl functional groups^43,44^. Furthermore, the intense band at ~3285 cm^−1^ can be assigned to typical –O-H stretching vibrations. The doublet at ~2896 and ~2826 cm^−1^ represents C-H stretching. The broad band at ~699 cm^−1^ was ascribed to =C–H stretching. The chemical composition and structure of the CQDs were analyzed with X-ray photoelectron spectroscopy (XPS) (Fig. S1). Two dominant peaks at ~286.14 eV (C 1s) and ~533.37 eV (O 1s) were observed in the XPS survey spectrum with elemental compositions of 71% and 29%, respectively. The high-resolution C 1s spectrum can be split into four peaks, as shown in Fig. 2(e). The peaks centered at ~284.4 eV, ~285.1 eV, ~286.4 eV, ~287.1 eV, and ~288.1 eV were assigned to C=C, C-C, C-O, C=O, and COO^−^, respectively^45^. The peaks at ~532.8 eV, ~531.9 eV, and ~535.2 eV, in the deconvoluted O 1 s spectrum of CQDs (Fig. 2(f)) were attributed to the binding of C-O, C=O, and COO^−^, respectively^46^.

**Figure 3:**
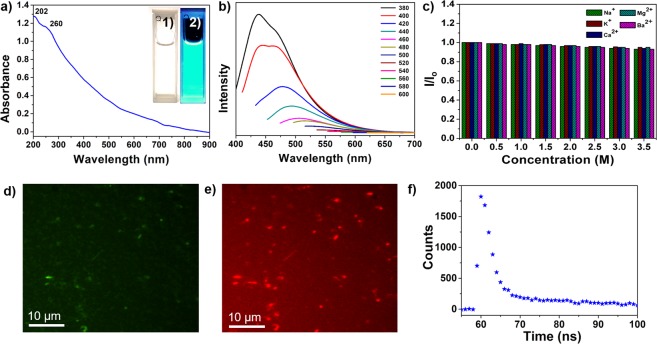
Optical properties of the CQDs. (**a**) UV-vis absorption spectrum; inset: digital photographs of an aqueous dispersion of CQDs under daylight (**1**) and 365 nm UV irradiation (**2**); (**b**) PL emissions with various excitation wavelengths from 380 to 600 nm; (**c**) the effect of the presence of different ions at various concentrations on emission intensities. PL microscopy images of a CQD solution evaporated on a glass slide under (**d**) 488 nm and (**e**) 562 nm band-pass filters. (**f**) PL decay profile of CQDs.

An aqueous dispersion of the CQDs was highly stable without any sign of precipitation even after a year. When irradiated with 365 nm UV light, the aqueous solution of CQDs showed an intense greenish blue color (inset of Fig. 3(a)), indicating its potential in the optoelectronic and biomedical fields. The CQDs show two absorption peaks at ~202 and ~260 nm (Fig. 3(a)) with a tail across the entire visible region, which is associated with several π−π^*^(C=C) and n−π^*^(C=O) transitions^47^. The PL emission of CQDs is dependent on the excitation wavelength, shifting to longer wavelengths with increases in the excitation wavelengths. The strongest emission was observed at 470 nm and shifted to 650 nm with a gradual decrease in intensity as the excitation wavelength was changed from 380 to 600 nm (Fig. 3(b)). The CQDs have negatively charged oxygenated carboxyl and hydroxyl functional groups as evidenced by the FTIR and XPS analyses, leading to discrete energy levels as emission centers. These emission centers are known to be responsible for the characteristic PL properties of CQDs via nonradiative recombination of charge carriers^18,48^. The tunable emissions of CQDs are thought to be due to the optical selection of distinct emissive traps on the surface^16^. The PL quantum yield of ~18% measured with reference to quinine sulfate falls within the previously report range for hydrothermally synthesized CQDs. No optical bleaching was observed under continuous irradiation at 365 nm for 60 min as shown in Fig. S2, which illustrates the excellent photostability of the CQDs. The dependence of the PL emissions of the CQDs on their chemical environment was investigated in the presence of a range of cations (Na^+^, K^+^, Ca^2+^, Mg^2+^, and Ba^2+^) at high ionic strength. The maximum intensity was almost unaffected by the presence of the different cations in a wide concentration range (Fig. 3(c)), confirming that the CQDs have considerable potential to be used as PL probes under high salt concentrations. PL images of the CQDs shown in Fig. 3(d,e) under 488 and 562 nm band-pass filters, respectively, confirm that the CQDs are highly fluorescent with tunable emissions. The time-resolved PL curve of CQDs measured at an excitation wavelength of 380 nm is shown in Fig. 3(f). The average radiative lifetime (*A*_τ_) of the charge carriers was estimated as 20.15 ns with a Chi^2^ value of 1.211, indicating an enhanced lifetime and charge carriers separation attributed to high density of surface functional groups on the CQDs^49^.

### Photocatalytic degradation of MB dye under visible-light irradiation

**Figure 4:**
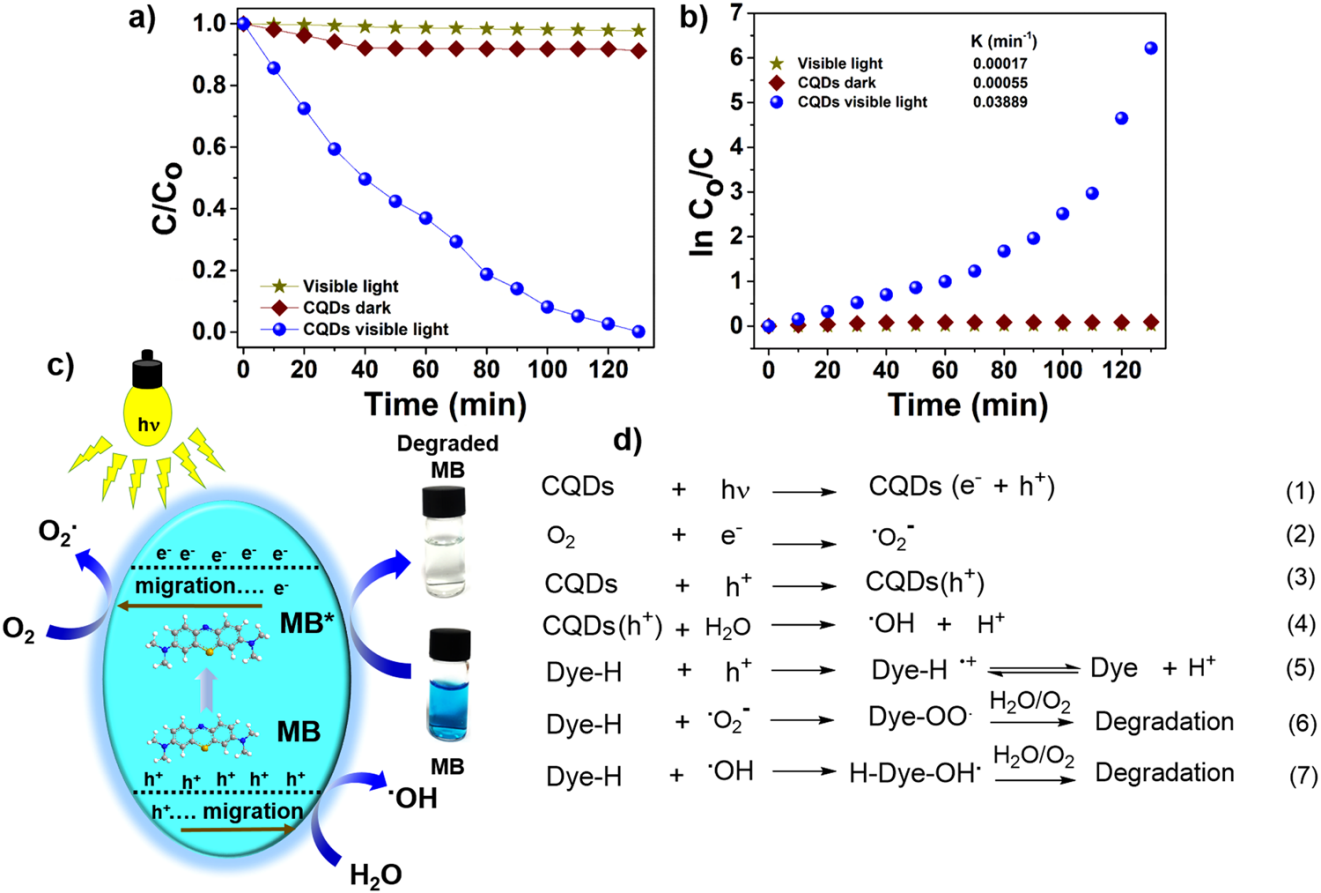
Visible-light-induced photodegradation of MB. (**a**) Photodegradation of MB with CQDs under different conditions; (**b**) plot of ln C_o_/C; (**c**) the proposed mechanism of MB degradation under visible-light irradiation; (**d**) proposed reactions involved in MB degradation.

The CQDs was used as a photocatalyst without conjugation to any semiconductor nanomaterial or heteroatom doping. Photodegradation of MB in aqueous solution was used to evaluate the photocatalytic performance of CQDs under visible light. The degradation of MB was evaluated by observing the change in UV-vis absorption with reaction time. The characteristic absorbance of MB centered at 664 nm did not show any decrease in intensity when irradiated with visible light in the absence of a catalyst, indicating that MB is resistant to visible-light irradiation (Fig. 4(a)). The CQDs in the dark led to only a slight decrease in MB concentration even after 130 min with a dye degradation efficiency of ~8.3% (Fig. 4(a)). In case of the CQDs under visible-light irradiation, a gradual decrease in MB concentration with time was observed with a photodegradation efficiency of ~99.5% within 130 min, indicating almost complete degradation. The inactivity of the CQDs towards MB in the dark confirms that the process is degradation rather than adsorption. The peak position of the characteristic MB absorption centered at 664 nm remains the same during the entire experiment with a significant decrease in intensity (Fig. S3), indicating the absence of any other chromophore molecules as a byproduct^50^. The apparent rate constant (k_app_) of MB degradation with CQDs was 0.03889 min^−1^ (Fig. 4(b)). The high photocatalytic efficiency of the CQDs can be explained on the basis of their visible-light absorption capacity and abundance of optically active centers. Enhanced light absorption of CQDs over the full visible light spectrum is likely to facilitate the generation of electron-hole (e^−^-h^+^) pairs. The surface traps on the CQDs could act as electron scavengers leading to suppressed electron-hole recombination and subsequent efficient charge transfer for the preferred photoreactions^50^. CQDs exhibited the PL lifetime of 20.15 ns, which further confirms that photoexcited e^−^-h^+^ pairs are efficiently separated. High density of separated h^+^ favors the formation of active radicals, which makes major contribution to the degradation of dye molecules^49^. The separation of e^−^-h^+^ pairs can be experimentally evident by PL quenching, where MB scavenges surface-trapped e^−^ or h^+^ and disturbs their radiative recombination^24^. The surface functional groups, such as -OH and -COOH, could act as the active sites for the photocatalytic reactions through efficient H-bonding interactions with MB dye molecules. In addition, fast electron transfer between the CQDs and surface-adsorbed MB could efficiently enhance the separation of (e^−^-h^+^) pairs^51^.

A plausible photocatalytic mechanism and the pathways for MB dye degradation are shown in Fig. 4(c,d). Photoexcitation of electrons from the valence band of the activated CQDs to the conduction band under visible-light irradiation leads to the generation of e^−^h^+^ pairs (Fig. 4(d), eq-1 and 3). Meanwhile, some e^−^ are trapped by surface defects, retarding the recombination of e^−^h^+^ pairs, and some are captured by the dissolved oxygen, generating superoxide radicals (^•^O_2_^−^) (Fig. 4(d), eq-2). The presence of ^•^O_2_^−^ with high activity results in the MB degradation (Fig. 4(d), eq-6). Photosensitized h^+^ either react with surface-adsorbed water molecules to form hydroxyl radicals (^•^OH) or directly interact with dye molecules to generate organic radicals (Fig. 4(d), eq-4 and 5). At the same time, the reactive oxygen species interact with MB molecules and degrade them (Fig. 4(d), eq-6 and 7).

**Figure 5:**
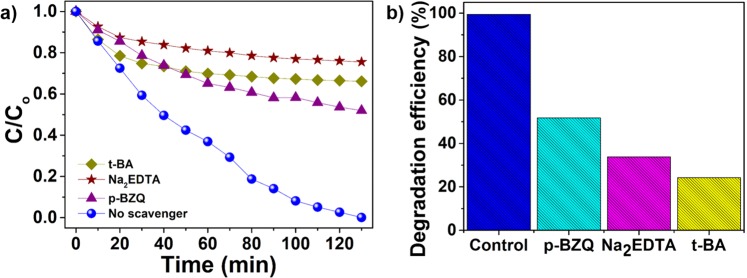
(**a**) Degradation of MB in the presence of different scavengers: Na_2_EDTA for holes, t-BA for hydroxyl radicals, and p-BZQ for superoxide radicals; (**b**) a comparison of the MB-degradation (130 min) performance of CQDs in the presence of the different scavengers.

In homogeneous aqueous medium, a photocatalyst under light irradiation usually generates three types of major active species; holes, superoxide, and hydroxyl radicals, which actively participate in photodegradation of dyes^52–54^. Active-species-trapping experiments were carried out to elucidate the roles of the major active species in the photocatalytic process and to explore the photocatalytic mechanism in greater depth. The individual application of 10 mM Na_2_EDTA (as h^+^ scavenger) and 10 mM *tert-*butanol (t-BA) (as ^•^OH scavenger) in the current system significantly suppressed the photodegradation of MB, which was attributed to the trapping of h^+^ and ^•^OH radicals, respectively (Fig. 5(a)). However, the introduction of para-benzoquinone (p-BZQ) (as ^•^O_2_^−^ scavenger) led to the smallest decrease in the MB degradation (Fig. 5(a)). The presence of Na_2_EDTA, t-BA, and p-BZQ suppressed the degradation efficiencies to ~25%, ~33% and ~50% (Fig. 5(b)), respectively, indicating that h^+^ and ^•^OH make the main contributions to the photocatalytic process, while ^•^O_2_^−^ is the secondary species. The structural advantages of the CQDs are considered to be their high density of surface traps and significant absorption of visible light leading to efficient photocatalytic activity, even under visible light.

### Fluorescence “turn-off-on” sensing of Fe(III) and AA

**Figure 6:**
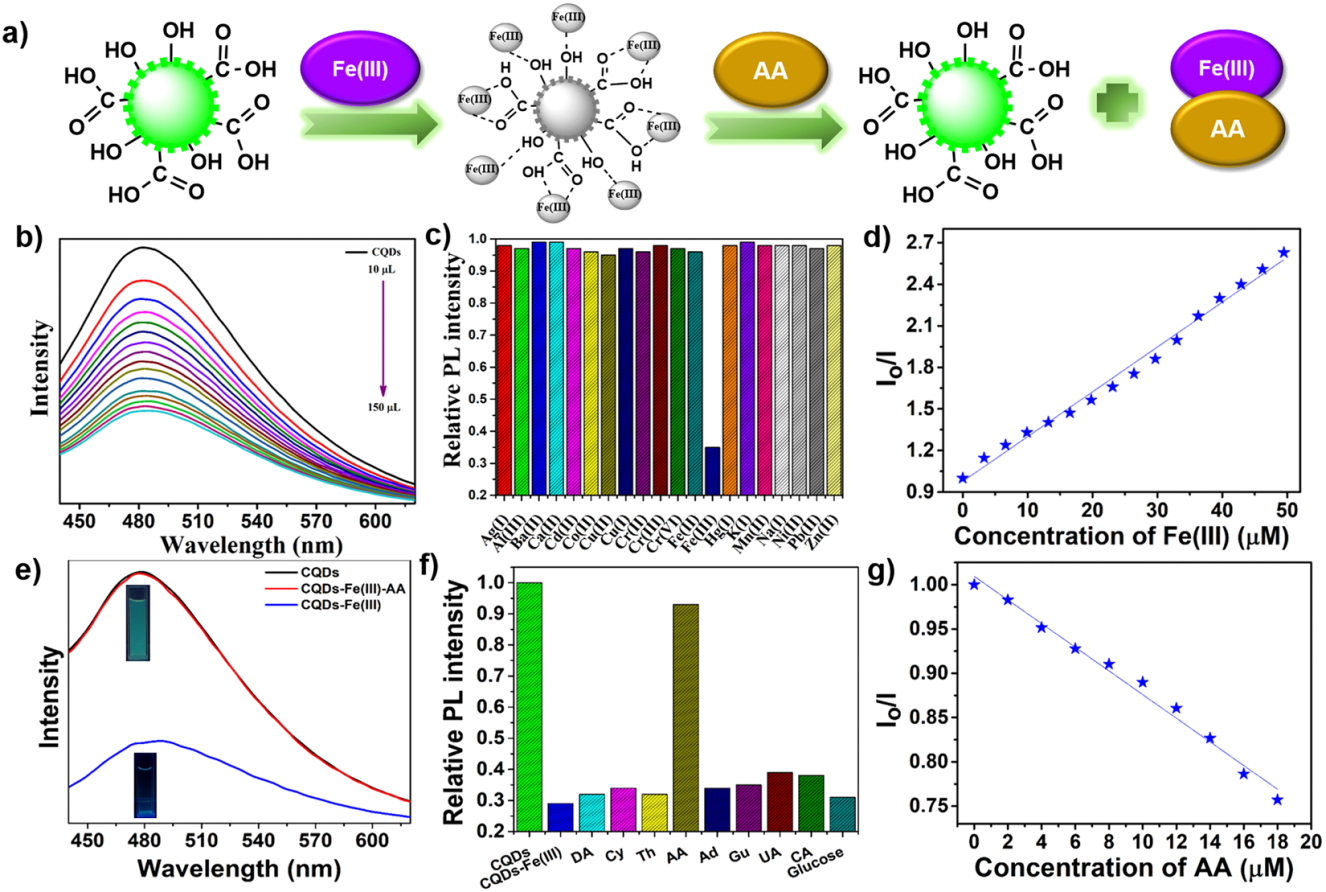
Selective detection of Fe(III) and AA. (**a**) Schematic illustration of the PL response of CQDs to Fe(III) and CQDs/Fe to AA; (**b**) PL emission spectra (λ_ex_ = 420 nm) of CQDs in the presence of Fe(III) with varying concentration; (**c**) comparison of PL intensity of CQDs in the presence of different metal ions; (**d**) Stern-Volmer plot for linear fitting of the PL intensity ratio versus the concentration of Fe(III) measured at 420 nm. I_o_ and I are the emission intensities of CQDs in the absence and presence of Fe(III), respectively (**e**) PL spectra of the CQDs/Fe system showing the restoration of emission intensity on the addition of AA; (**f**) comparison of PL “turn-on” emission behavior upon the addition of different biomolecules to the quenched CQDs/Fe(III) system; (**g**) relationship between PL intensity restoration and AA concentration.

The feasibility of using CQDs as efficient PL probes was explored in the selective detection of Fe(III) and AA. A schematic illustration of the quenching and restoration of CQDs’ PL upon addition of Fe(III) and AA, respectively, is shown in Fig. 6(a). Fe(III) selectively affected the emission of CQDs, leading to the quenching of their PL (Fig. 6(a)), which can be attributed to the binding of Fe(III) to surface functional groups on the CQDs. Gradual addition of Fe(III) (1 × 10^−3^ M) in varying amounts (10 µL to 150 µL) resulted in rapid and efficient quenching of the PL emission centered at 472 nm without any change in the peak position, as shown in Fig. 6(b). The selectivity of the CQDs to Fe(III) was evaluated with interference assays with various metal ions to determine the practicality of using the CQDs in complex solutions. As shown in Fig. 6(c), except for Fe(III), none of the metal ions caused a notable decrease in the PL intensity, clearly demonstrating that the CQDs exhibited good selectivity toward Fe(III). The selective PL quenching of the CQDs by Fe(III) exhibited a linear relationship with Fe(III) concentration, as shown in Fig. 6(d). The detection limit for Fe(III) was calculated according to 3δ/m (δ denotes the standard deviation and m represents the slope of the linear fit) to be 2.28 µM with a correlation coefficient of 0.989^51^. The selectivity towards Fe(III) was attributed to the ion-selective nature of the oxygenated functional groups and enhanced charge-transfer effects. The Fe(III) interacted with oxygenated functional groups on the CQDs leading to the formation of complexes, which changed the distribution of the energy states and enhanced the nonradiative recombination of charge carriers, subsequently resulting in PL quenching^51^.

The zeta potential of the CQDs changed from −36.8 eV to −11.2 eV after the addition of 150 µL of Fe(III), revealing the successful binding of Fe(III) to the CQDs through electrostatic interactions between the positively charged Fe ions and negatively charged CQDs. More significantly, the “turn-on” emission behavior of the quenched CQDs/Fe(III) system was selectively activated by the addition of AA. “Turn-on” probes are more sensitive and selective than “turn-off” ones but are currently less common. The addition of AA (18 µM) to the completely quenched CQDs/Fe(III) system led to immediate PL recovery (Fig. 6(e)), demonstrating “turn-on” behavior. The PL restoration was attributed to the release of loosely bound Fe(III) from the surfaces of the CQDs^31^. Under UV illumination at 365 nm, the colorless CQDs/Fe(III) system changed to greenish blue as shown in Fig. 6(e).

As a necessary step toward determining the practical applicability of the CQDs, their selectivity to AA was explored against several endogenous biomolecules including dopamine (DA), cytosine (Cy), thymine (Th), adenine (Ad), guanine (Gu), uric acid (UA), and citric acid (CA). Only AA led to a remarkable enhancement in PL emission, as shown in Fig. 6(f). The selectivity of the CQDs/Fe(III) system to AA can be explained on the basis of favorable interactions between AA and Fe(III) owing to their high binding affinity. A linear calibration plot of the intensity ratio at different concentrations of AA with a correlation coefficient of 0.986 is presented in Fig. 6(g). The PL-emission intensity ratio of CQDs/Fe(III) gradually increased with the addition of AA and a detection limit of 1.27 µM was obtained based upon the expression 3δ/m.

## Conclusions

Visible-light-sensitive CQDs were synthesized by a facile hydrothermal process using pear juice as the carbon source. The structural advantages of the CQDs, their surface functional groups and high density of surface defects, allowed their multifunctional application as a photocatalyst and PL probe. The CQDs showed highly effective visible-light-induced photocatalytic activity leading to almost complete photodegradation of MB dye; within 130 min, 99.5% of the dye had been degraded under visible light. The main reactive species were determined to be holes and hydroxyl radicals, and a plausible mechanistic pathway was proposed on the basis of radical-trapping experiments. Moreover, the as-synthesized CQDs were also explored as PL probe for the selective and sensitive detection of Fe(III) and AA. The CQDs could serve as a promising platform for multifunctional photomediated applications and sensing devices.

## Supplementary information

Supplementary Information
