# Supplementary material for: Biomass-derived Carbon Quantum Dots for Visible-Light-Induced Photocatalysis and Label-Free Detection of Fe(III) and Ascorbic acid
Source: Sci Rep. 2019 Oct 21;9:15084. doi: 10.1038/s41598-019-49266-y (PMC6803716; doi:10.1038/s41598-019-49266-y)
Supplement: Supplementary file 1 — Supplementary Information [file 41598_2019_49266_MOESM1_ESM.pdf]

Supplementary Information

Biomass-derived Carbon Quantum Dots for Visible-Light-Induced Photocatalysis and Label-Free Detection of Fe(III) and Ascorbic acid

Gouri Sankar Das[a], Jong Pil Shim[b], Amit Bhatnagar[c], Kumud Malika Tripathi[*a], and TaeYoung Kim[*a]

[a] *Department of Bionanotechnology, Gachon University, 1342 Seongnam-daero, Sujeong-gu Seongnam-si, Gyeonggi-do 13120, South Korea*

[b] *Department of Materials Science and Engineering, Korea University, 145 Anam-ro, Seongbuk-gu, Seoul 02841, Korea*

[c] *Department of Environmental and Biological Sciences, University of Eastern Finland, FI-70211, Kuopio, Finland*

[d] *Department of Materials Science and Engineering, Gachon University, 1342 Seongnam-daero, Sujeong-gu Seongnam-si, Gyeonggi-do 13120, South Korea*

Corresponding Author: kumud20010@gmail.com, taeykim@gachon.ac.kr

Chemical composition of CQDs

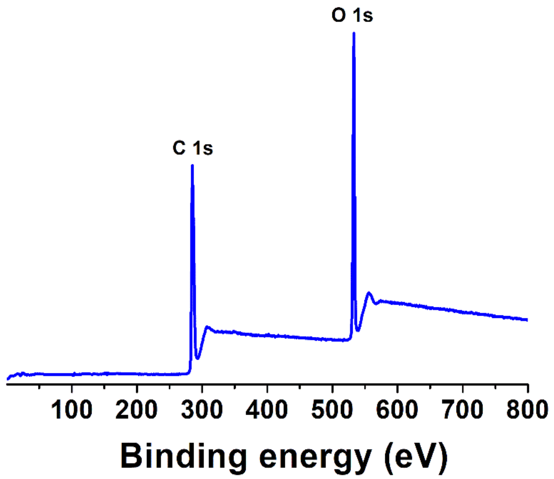

Figure S1. Wide-angle XPS spectrum of CQDs.

Table S1. EDS analysis of CQDs.

Element	Weight%	Atomic %
C K	73.22	78.45
O K	26.78	21.55
Totals	100.00	

Photostability of CQDs

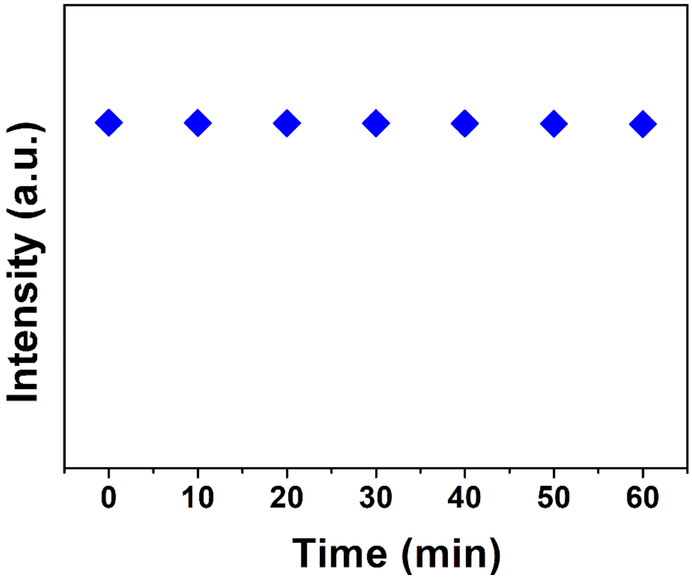

Figure S2. PL emission of CQDs under continuous UV (365 nm) irradiation for 60 min.

Photocatalytic degradation of MB dye under visible-light irradiation

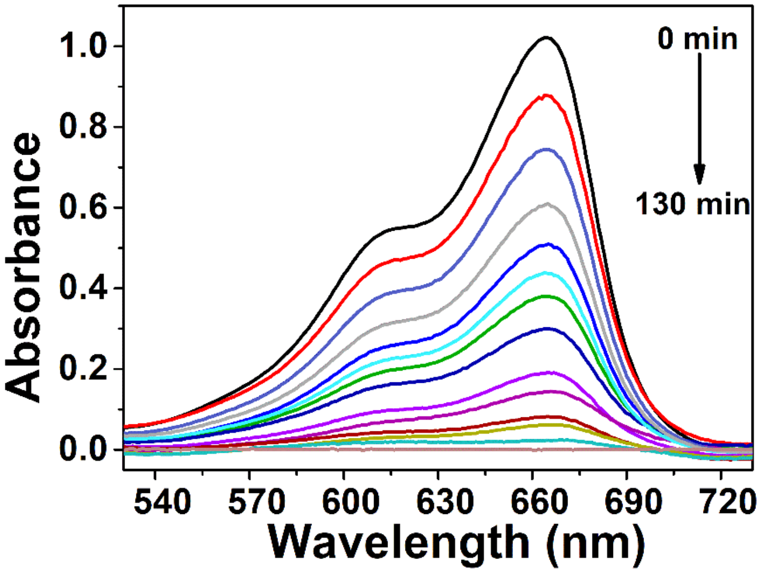

Figure S3. UV-vis absorption spectra of MB in presence of CQDs under visible light irradiation;
